# Effect of Mutated Cu, Zn Superoxide Dismutase (SOD1^G93A^) on Modulation of Transductional Pathway Mediated by M1 Muscarinic Receptor in SK-N-BE and NSC-34 Cells

**DOI:** 10.3389/fphys.2018.00611

**Published:** 2018-05-24

**Authors:** Simona Damiano, Anna Sasso, Roberta Accetta, Marcellino Monda, Bruno De Luca, Luigi Michele Pavone, Anna Belfiore, Mariarosaria Santillo, Paolo Mondola

**Affiliations:** ^1^Dipartimento di Medicina e Chirurgia, Università di Napoli Federico II, Naples, Italy; ^2^Dipartimento di Medicina Sperimentale, Università degli Studi della Campania “Luigi Vanvitelli”, Naples, Italy; ^3^Dipartimento di Medicina Molecolare e Biotecnologie Mediche, Università di Napoli Federico II, Naples, Italy

**Keywords:** wild type superoxide dismutase, G93A superoxide dismutase, P-ERK, P-AKT, M1 muscarinic receptor

## Abstract

The constitutive secretion of antioxidant Cu-Zn Superoxide dismutase (SOD1) has been widely demonstrated in many cellular lines. In addition, we showed that as well as the basal SOD1 secretion, this enzyme is also exported through depolarization of excitable cells by high extracellular K concentration. Recent data showed that SOD1 was able to activate muscarinic M1 receptor producing the activation, via phospholipase C, of ERK1-2 and AKT pathways. It is also known that about 20% of familial amyotrophic lateral sclerosis (fALS) is due to mutations in the gene coding for SOD1. The aim of the present research is to evaluate whether, analogously to wild type SOD1 (SOD1^wt^), the mutated form of SOD1^G93A^ is able to activate ERK1-2 and AKT through muscarinic M1 receptor in SK-N-BE as well as in motoneuron like NSC-34. Our results demonstrated that in NSC-34 and SK-N-BE cells mutated SOD1^G93A^ carried out a more evident activation of ERK1-2 and AKT and a stronger increase of intracellular calcium levels compared to SOD1^WT^; we also demonstrated that these effects are mediated by the M1 receptor as shown using pirenzepine, a specific M1 inhibitor and the calcium chelator BAPTA. Of note, M1 receptor pathway activation by SOD1G93A, but not by SOD1WT, is associated with both an increase of reactive oxygen species and a cytotoxic effect.

## Introduction

Cu, Zn superoxide dismutase (SOD1) is a dimeric ubiquitous enzyme mainly localized in the cytosol that catalyzes the dismutation of superoxide radical into hydrogen peroxide and molecular oxygen; in association with non-enzymatic antioxidants it plays a pivotal role in the cellular homeostasis of reactive oxygen species (ROS) (Fridovich, 1995; Russo et al., 2011). It has been shown that free radicals are not only byproducts of the cellular metabolic utilization of oxygen but also represent signaling molecules regulating multiple functions (Damiano et al., 2012, 2015; Santillo et al., 2015; Accetta et al., 2016).

SOD1 secretion has been demonstrated in many cellular lines (Mondola et al., 1996, 1998; Cimini et al., 2002; Terrazzano et al., 2014). It is known that the export of this enzyme in SK-N-BE human neuroblastoma cells is ATP-dependent (Mondola et al., 2003) and involves microvesicles (Gomes et al., 2007). In addition, we showed that depolarization, caused by high extracellular K^+^ concentrations, induces an additional inducible calcium-dependent SOD1 secretion through T and V SNARE complexes in excitable GH3 rat pituitary cells (Santillo et al., 2007). Moreover, this enzyme, through the activation of a calcium dependent pathway, can participate in various biological functions (Mondola et al., 2002; Viggiano et al., 2012). However, the mechanism involved in the molecular secretory pathway of SOD1 is not yet clear (Mellman and Warren, 2000; Bosco et al., 2010; Forsberg et al., 2010).

Mutant SOD1 endoplasmic reticulum/Golgi transport of SOD1 in amyotrophic lateral sclerosis (ALS) has also been demonstrated (Atkin et al., 2008). Previously, we showed that SOD1 interacts with the membrane cell of SK-N-BE activating phospholipase C with subsequent increase of intracellular calcium concentrations (Mondola et al., 2004).

Recent *in vitro* data performed in SK-N-BE cells showed that SOD1 was able to activate muscarinic M1 receptor producing an activation of ERK1-2 and AKT, in a dose and time-dependent manner, accompanied with an increase of intracellular calcium concentrations (Damiano et al., 2013). Recently, we highlighted new function of SOD1 pointing out some inedited effects carried out by this enzyme in addition to its classic function of oxygen radical dismutase (Mondola et al., 2016).

Amyotrophic lateral sclerosis is a progressive neurodegenerative disease determined by loss of motor neurons (Rowland and Shneider, 2001). It is known that 15–20% of familial ALS (fALS) cases are due to mutations of SOD1 gene. However, the neurotoxic mechanism of SOD1 mutants involved in fALS still remains unclear (Bosco et al., 2010; Forsberg et al., 2010). Indeed, more than 110 mutations in this gene have been described; in addition, many SOD1 mutants retain their enzymatic activity suggesting the possibility of toxic function gain of these forms of mutated SOD1 in fALS (Strong et al., 2005; Dion et al., 2009). Motoneuron like NSC-34 cells represent a good experimental model for the study of ALS because they maintain the morphological and functional characteristics of motor neurons as cell body, dendrites and axon terminals (Turner et al., 2005).

The aim of the present study is to evaluate whether, similarly to wild type SOD1, SOD^G93A^, that represents the mutated SOD1 mostly used for *in vivo* (Gurney, 1994) and *in vitro* (Sun et al., 2013) studies of human fALS, is able to activate ERK1-2 and AKT through the muscarinic M1 receptor in both SK-N-BE and motoneuron like NSC -34 cells. In addition, we investigated the involvement of M1 receptor on the cytotoxic effect of SOD^G93A^.

## Materials and Methods

### Reagents

SOD1^wt^, Carbamoylcholine chloride (Cch) and Pirenzepine were purchased from Sigma-Aldrich (St. Louis, MO, United States). BAPTA-AM was purchased from Calbiochem (United States). Mutated form, SOD1^G93A^, was provided by the “Recombinant Protein Service” of PRIMM srl (Milano, Italy).

### Cell Cultures

*SK-N-BE cells* are human neuroblastoma cells (CELLution Biosystem Inc., Canada).

These cells were grown in Dulbecco’s Modified Eagles Medium (RPMI 1640; GIBCO Invitrogen) supplemented with 10% FBS (Sigma S. Louis, United States), 2 mM L-glutammina, 100 U/ml penicillin and 100 μg/ml streptomycin.

*MO3-13 cells* (CELLution Biosystem Inc., Canada), an immortal human–human hybrid cell line, were grown in Dulbecco’s Modified Eagles Medium (DMEM; GIBCO Invitrogen), containing 4.5 g/L glucose (GIBCO, Auckland, New Zealand), supplemented with 10% Fetal Bovine Serum (FBS; Sigma S. Louis, United States), 2 mM L-glutammina,100 U/ml penicillin and 100 μg/ml streptomycin.

NSC-34 cells kindly supplied by Dr. Cashman (University of Toronto, Toronto Ontario Canada) are neuroblastoma-spinal motor neuron fusion cells that represent a good model for the study of ALS. The cells were grown in Modified Eagles Medium (MEM; GIBCO Invitrogen), containing 4.5 g/L glucose (GIBCO, Auckland, New Zealand), supplemented with 10% FBS (Sigma S. Louis, United States), 100 U/ml penicillin and 100 μg/ml streptomycin.

The cells were kept in a 5% CO**_2_** and 95% air atmosphere at 37°C before use.

### Presence of Muscarinic M1 Receptor in NSC-34 Cells

Human neuroblastoma SK-N-BE, oligodendrocyte MO3-13 and NSC-34 cells were grown to semiconfluence in 35 mm culture dishes in complete RPMI medium (SK-N-BE), MEM medium (NSC-34) and DMEM medium (MO3-13) and were then incubated for 18h in a medium containing 0.2% FBS and collected by scraping into a RIPA buffer (50 mM Tris–HCl, pH 7.5, 150 mM NaCl, 1% NP40, 0.5% deoxycholate, 0.1% SDS, 2.5 mM Na-pyrophosphate, 1 mM β-glycerophosphate, 1 mM NaVO_4_, 1 mM NaF, 0.5 mM PMSF and a cocktail of protease inhibitors Roche, United States).

The cells were disrupted by repeated aspiration through a 21-gage needle. The supernatants were then centrifuged at 13000 rpm for 15 min at 4°C to remove nuclei and any intact cells and were collected to determine the protein concentration according to the Lowry method (Lowry et al., 1951). Electrophoresis of cellular lysates was performed according to the protocol proposed by Laemmli (1970) using as reference a mixture of proteins of known molecular weight (Invitrogen, Sharp^®^ Novex Pre-Stained Protein Standards). Fifty micrograms of total proteins were processed by 10% SDS-polyacrylamide gel (SDS–PAGE). Filters were incubated with rabbit polyclonal antibodies M1 muscarinic receptor purchased by Sigma (United States) diluted 1:1000 in TBST 0.1% and then incubated with a peroxidase-conjugated secondary antibody diluted 1:2000 in TBST 0.1% for 1 h at room temperature (GE-Healthcare, United Kingdom). Peroxidase activity was detected with the ECL system (GE-Healthcare, United Kingdom). Protein bands were quantified by densitometry using Scion Image software. Densitometric values were normalized with mouse monoclonal α-tubulin antibodies diluted 1:3000 for 1 h at room temperature and then incubated with a peroxidase-conjugated secondary antibody diluted 1:2000 in TBST 0.1% for 1 h at room temperature (Sigma, United States). Each experiment was repeated three times (Mondola et al., 1994; Damiano et al., 2013).

### Preparation of SK-N-BE and of NSC-34 Cells Lysates and Western Blotting Analysis for Phosphorylated Proteins P-ERK1-2 and P-AKT

The cell models of mice motor neurons (NSC-34) were grown until 70% confluence in 35 mm Petri dishes and incubated for 18 h with medium containing 0.2% FBS. The cells were incubated with 400 ng/ml of SOD1 at various times (10 and 30 min) and then washed with phosphate buffer salts (phosphate-buffered saline, PBS) and lysed in RIPA buffer.

Fifty micrograms of total protein were loaded on a 10% polyacrylamide gel and transferred on two nitrocellulose membranes by Western Blotting, with a procedure similar to that previously indicated. The membrane was placed in blocking solution, 5% non-fat milk in tris-buffered saline, 0.1% Tween 20 (TBST), at 4°C for 2 h to block the non specific binding sites. The filter was cut in two parts, each of which incubated with specific antibodies against anti-phosphorylated ERK1-2 (P-ERK1-2, Santa Cruz Biotechnology Inc.) and anti-phosphorylated AKT (P-AKT, Santa Cruz Biotechnology Inc.) respectively. After appropriate washes in TBST 0.1%, the membranes were incubated for 1 h with a peroxidase-conjugated secondary antibody (GE-Healthcare, United Kingdom.). The detection of the immunoreactive bands was performed by chemiluminescence with ECL (enhanced chemiluminescence, Western Blotting Analysis System, Amersham Biosciences UK Limited). The quantitative analysis was carried out by densitometry, after incubating the membranes with rabbit polyclonal antibodies anti total ERK and anti-total AKT respectively diluted 1:1000 in TBST 0.1% and incubated for 1 h at room temperature, and then incubated with a peroxidase-conjugated secondary antibody diluted 1:2000 in TBST 0.1% for 1 h at room temperature (Santa Cruz Biotechnology Inc.).

### Analysis of ERK1-2-AKT-Dependent Transduction Pathway in Presence of SOD1^wt^ and SOD1^G93A^ of SK-N-BE and of NSC-34 Cells

SK-N-BE and NSC-34 cells were grown until 70% confluence in 35 mm Petri dishes and incubated for 18 h with medium (RPMI and MEM respectively) containing 0.2% FBS. The cells were incubated either with 400 ng/ml of SOD1^wt^ or with 400 ng/ml of mutated form of SOD1 (SOD1^G93A^) for 30 and 60 min respectively in presence and absence of 15 μM pirenzepine, an antagonist of M1 muscarinic receptor. Cells were then washed with phosphate buffer salts (phosphate-buffered saline, PBS) and lysed in RIPA buffer.

Fifty micrograms of total protein were loaded on a 10% SDS-polyacrylamide gel (SDS–PAGE) and transferred on two nitrocellulose membranes by Western Blotting. The membranes were then treated as previously described.

### Cell Viability

The viability of SK-N-BE and NSC-34 cells was evaluated both by the determination of cleaved form (89 KDa) of poly ADP-ribose polymerase-1 (PARP-1) used as a marker for apoptosis evaluation and by Trypan Blue assay. Briefly, semiconfluent cells were preincubated with pirenzepine, a specific M1 receptor antagonist and intracellular calcium chelator BAPTA-AM for 30 and 5 min respectively and were then incubated for 4h in presence and in absence of 400 ng/ ml SOD1^wt^ or mutated SOD1^G93A^. Electrophoresis of 50 μg of cell proteins was performed as described above.

The membranes were incubated with a primary polyclonal anti rabbit PARP-1 antibody diluted 1:1000 in TBST 0.1% overnight at 4°C (Santa Cruz Biotechnology Inc.). Then, both membranes were incubated with peroxidase-conjugated secondary antibody diluted 1:2000 for 1 h at room temperature (GE-Healthcare, United Kingdom). The detection of the immunoreactive bands was performed by chemiluminescence with ECL (enhanced chemiluminescence, Western Blotting Analysis System, Amersham Biosciences UK Limited). The quantitative analysis was carried out by densitometry, after incubating the membranes with an anti-α-tubulin antibody from murine serum (Santa Cruz Biotechnology Inc.).

A further evaluation of cell viability in both cellular lines was performed by Trypan Blue assay. Briefly both cell lines incubated as previously described, were suspended in diluted trypan blue (1:1 with PBS) and then immediately counted in Burker’s chamber.

### Intracellular Calcium Determination

Intracellular calcium levels in SK-N-BE and NSC-34 cells were measured using the Fluo-4 NW calcium indicator kit (Life Technologies), according to the manufacturer’s instructions. Briefly, culture adherent cells starved for 18 h in 96 well microplates (20000/w) were washed with assay buffer and loaded with Fluo-4 probe in the presence of 5 mM Probenecid for 30 min at 37°C and then for further 30 min at room temperature. Fluorescence was recorded every 10 s using the Flouroskan Ascent–FL (Thermo electronic corporation) with excitation at 485 nm and emission at 538 nm. Fluorescence was recorded for 10 s (t0) before loading cells with 400 ng/ml SOD1^wt^ and SOD1^G93A^; and was then monitored for an additional 60 s. Blank samples fluorescence was also measured and subtracted from all experimental points.

### Fluorimetric Determination of Reactive Oxygen Species (ROS)

Fluorimetric determination of ROS levels were determined by the membrane-permeant ROS sensitive fluorogenic probe 5,6-carboxy-2,7 dichlorofluorescein diacetate, DCHF-DA (Molecular Probes, Leiden, The Netherlands). SK-N-BE and NSC34 cells were grown to semi-confluence in 24 multiwell plates and then incubated for 18 h in medium containing 0.2% FBS before the experiments. Cells were incubated for 30 min with SOD1^wt^ and mutated SOD1^G93A^. The cells were washed twice with PBS and incubated in the same buffer with 10 μM DCHFDA for 10 min. The cells were washed three times with PBS containing 10 mM glucose, 1.2 mM MgCl2 and 1.2 mM CaCl2 and dichlorofluorescein (DCF) fluorescence was measured at different time intervals using the plate reader Fluoroskan Ascent FL fluorometer (Thermo Electron Oy, Vantaa, Finland) and data was analyzed by Ascent software.

### Statistical Method for Data Analysis

The data shown are mean ± standard error (SE). Statistical analyses were performed using Student *t*-test for unpaired samples. Statistical differences in kinetic experiments were evaluated using one-way ANOVA followed by a Bonferroni *post hoc* test. *P*-values less than 0.05 were considered statistically significant. All analyses were performed using MedCalc program.

## Results

Previous studies, in our research laboratory, demonstrated that SOD1 interacts specifically with the cell surface of SK-N-BE activating M1 muscarinic receptor, and the downstream cascade of reactions leading to the ERK1-2 and AKT phosphorylation and subsequent increase of intracellular calcium concentrations (Damiano et al., 2013).

**FIGURE 1:**
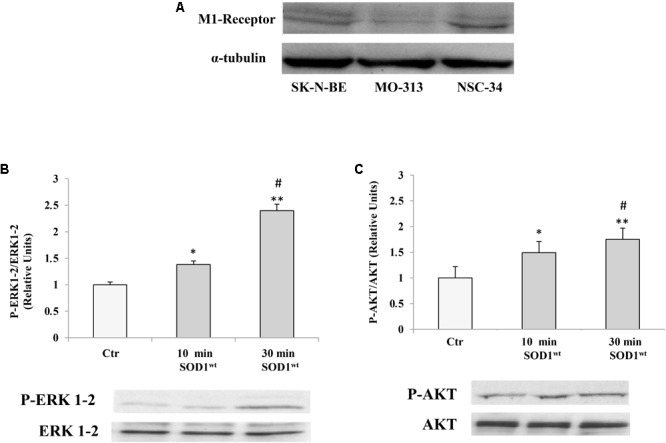
**(A)** Presence of muscarinic acetylcholine M1 receptor in human neuroblastoma SK-NBE, oligodendrocytes MO3-13 and neuroblastoma-spinal motor neuron fusion cells NSC-34 M1 receptor protein bands normalized to α-tubulin. Effect of SOD1 on ERK1-2 **(B)** and AKT **(C)** activation in NSC-34 cells incubated with 400 ng/ml of SOD1 for 10 and 30 min. The data represent the means ± SEM of three independent experiments relative to control obtained by densitometric analysis of P-ERK1-2 and P-AKT protein bands normalized to ERK1-2 and AKT, respectively. ^∗^*p* < 0.05 vs. Ctr; ^∗∗^*p* < 0.001 vs. Ctr; ^#^*p* < 0.001 vs. SOD1 10 min.

Therefore, it was verified that M1 muscarinic receptor was expressed in different cells of the nervous system like NSC-34 and MO3-13 (Human Oligodendrocytes Cell Line) as well as in SK-N-BE **Figure 1A**.

### SOD1 Activates a Transduction Pathway That Involves P-ERK1-2 and P-AKT in NSC-34 Cells

We used NSC-34 cells to verify modulatory effects of SOD1 on important transduction pathways involving phosphorylated protein P-ERK1-2 and P-AKT.

**Figures 1B,C** shows time-course experiments, in which the cells were incubated at 37°C for 10 and 30 min with 400 ng/ml of SOD1. Western Blotting analysis showed a significant increase of P-ERK1-2 and P-AKT levels already after 10 min compared to controls.

The values for the P-ERK1-2 and P-AKT, obtained from the average of three independent experiments, were normalized for total ERK1-2 and total AKT, respectively.

### Activation of PKC-ERK1-2-AKT-Dependent Transduction Pathway by SOD1^G93A^ in SK-N-BE and in NSC-34 Cells

We used SK-N-BE and NSC-34 cells to verify modulatory effects of SOD1^wt^ compared with SOD1^G93A^ on phosphorylated protein P-ERK1-2 and P-AKT.

**FIGURE 2:**
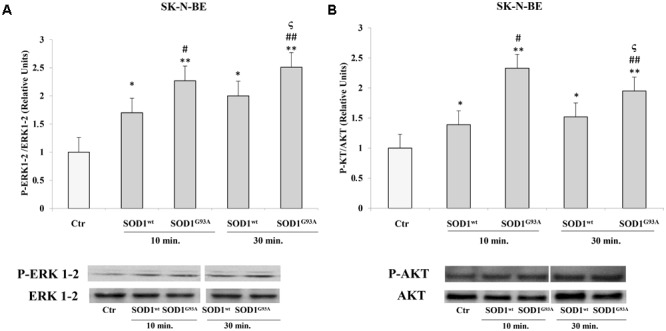
Activation of ERK1-2 and AKT trasductional pathways in presence of SOD1^wt^ and SOD1^G93A^ in SK-N-BE cells. Western blotting analysis of P-ERK **(A)** and P-AKT **(B)** in cells incubated with 400 ng/ml of SOD1^wt^ and SOD1^G93A^ for 10 and 30 min. The histograms show the mean values (+SE) evaluated by densitometric analysis of three independent experiments. The results were normalized to ERK1-2 and AKT respectively. ^∗^*p* < 0.05 vs. Ctr; ^∗∗^*p* < 0.001 vs. Ctr; ^#^*p* < 0.05 vs. 10 min SOD1^wt^; ^##^*p* < 0.001 vs. 10 min SOD1^wt^; ^ς^*p* < 0.05 vs. 30 min SOD1^wt^.

**Figures 2A,B** shows the time-course experiments, in which SK-N-BE cells were incubated at 37°C for 10 and 30 min with 400 ng/ml of SOD1**^wt^** or with SOD1^G93A^, at the same concentration. The data obtained revealed a significant increase of P-ERK1-2 and P-AKT levels already after 10 min in presence of SOD1^G93A^ compared to cells incubated with SOD1^wt^.

**FIGURE 3:**
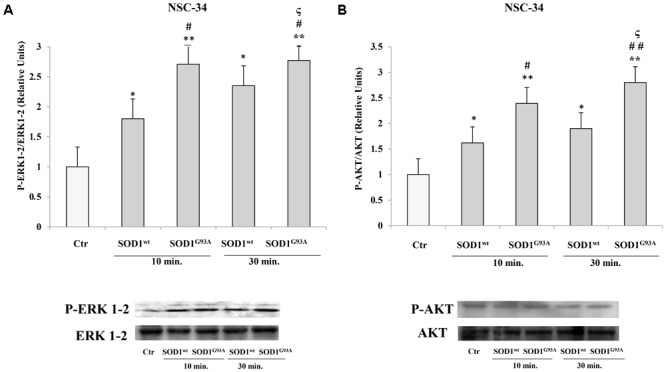
Activation of PLC-PKC-ERK1-2-AKT-dependent transduction pathway in presence of SOD1^wt^ and SOD1^G93A^ in NSC-34 cells. Western Blotting analysis of the levels of P-ERK 1-2 **(A)** and P-AKT **(B)** in NSC-34 cells incubated with 400 ng/ml of SOD1^wt^ and with 400 ng/ml of SOD1^G93A^ for times of 10 and 30 min. The data represent the means ± SEM of three independent experiments relative to control obtained by densitometric analysis of P-ERK1-2 and P-AKT protein bands normalized to ERK1-2 and AKT, respectively. ^∗^*p* < 0.05 vs. Ctr; ^∗∗^*p* < 0.001 vs. Ctr; #*p* < 0.05 vs. 10 min SOD1^wt^; ^##^*p* < 0.001 vs. 10 min SOD1^wt^; ^ς^*p* < 0.05 vs. 30 min SOD1^wt^.

In NSC-34 cells, likewise SK-N-BE cells, SOD1^wt^ and SOD1^G93A^ increased P- ERK1-2 and P-AKT levels; in addition, in cells incubated with SOD1^G93A^ mutant the phosphorylation levels of the two proteins were significantly higher compared to those of cells incubated with SOD1^wt^ **Figures 3A,B**.

### Effect of Muscarinic M1 Receptor Antagonist on Activation of ERK1-2 and AKT Pathways in SK-N-BE and NSC-34 Cells by SOD1^G93A^

**FIGURE 4:**
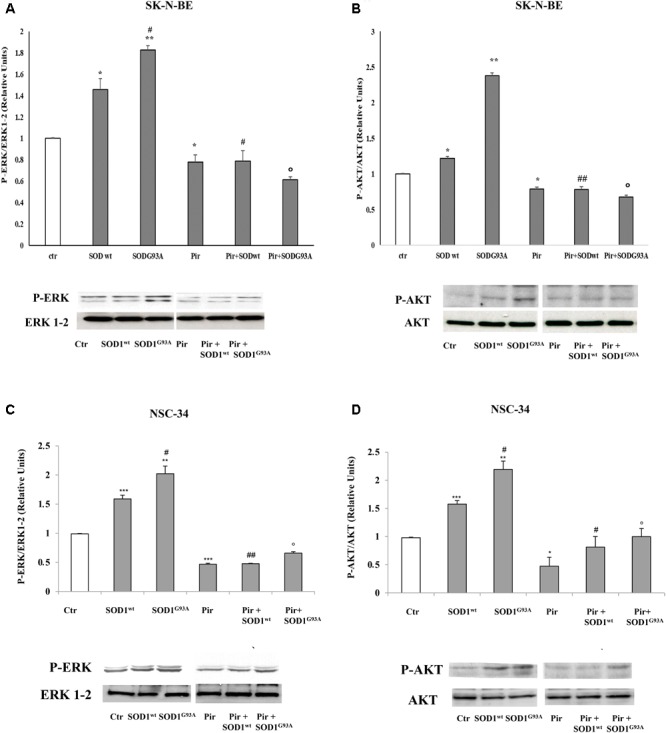
Activation of PLC-PKC-ERK 1-2-AKT-dependent transduction pathway in presence of SOD1^wt^ and SOD1^G93A^. Western Blotting analysis of the levels of P-ERK 1-2 and P-AKT in SK-N-BE **(A,B)** and NSC-34 **(C,D)** cells incubated for 10 min with 400 ng/ml of SOD1^wt^ and with 400 ng/ml of SOD1^G93A^ in absence and in presence of Pirenzepine 10 μM for 5 min. The data represent the means ± SEM of three independent experiments relative to control obtained by densitometric analysis of P-ERK1/2 and P-AKT protein bands normalized to ERK1-2 and AKT respectively. ^∗^*p* < 0.05 vs. Ctr; ^∗∗^*p* < 0.01 vs. Ctr; ^∗∗∗^*p* < 0.001 vs. Ctr; ^#^*p* < 0.05 vs. SOD1^wt^; ^##^*p* < 0.001 vs. SOD1^wt^; °*p* < 0.01 vs. SOD1^G93A^.

Previously, (Damiano et al., 2013) we demonstrated the inhibitory effect of pirenzepine on SOD1-dependent PLC/ERK1-2/AKT pathway activation mediated by muscarinic M1 receptor in SK-N-BE cells. In **Figure 4**, we demonstrated that the preincubation of SK-N-BE **(A,B)** and NSC-34 **(C,D)** cells with 15 μM of pirenzepine, a specific M1 receptor antagonist (Roldán et al., 1997; Saygi Bacanak et al., 2017), in the same experimental conditions reverts the stimulatory effect of SOD^G93A^ on ERK1-2 and AKT levels. This data highlights the involvement of muscarinic M1 receptor in the ERK1-2/AKT pathway activation by mutated SOD1.

### Intracellular Calcium, ROS and Cell Viability Determination

**FIGURE 5:**
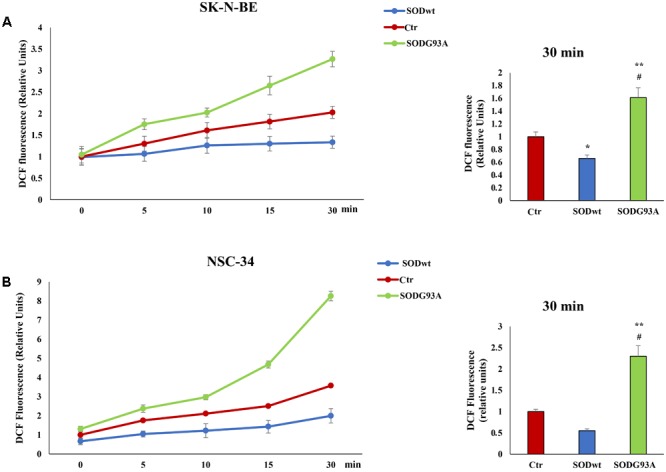
Fluorimetric Determination of Reactive oxygen species (ROS) levels. SK-N-BE **(A)** and NSC-34 **(B)** semiconfluent cells were incubated for 18 h in medium containing 0.2% FBS and then for 30 min with 400 ng/ml SOD1^wt^ and mutated SOD1^G93A^. To determine Reactive Oxygen Species (ROS) levels, SK-N-BE **(A)** and NSC-34 **(B)** cells were incubated with 10 μM of the ROS sensitive probe DCHF-DA and ROS levels were measured by fluorometric analysis. The graphs on the left show the mean ± SEM values (*n* = 6) relative to control of the indicated time points. The histograms on the right show the values of 30 min point and the relative ANOVA analysis. ^∗^*p* < 0.05 vs. Ctr; ^∗∗^*p* < 0.001 vs. Ctr; ^#^*p* < 0.001 vs. SOD^wt^.

Reactive oxygen species are involved in many neurodegenerative diseases (Damiano et al., 2012; Damiano et al., 2015; Accetta et al., 2016) therefore we performed additional experiments evaluating ROS production in SK-N-BE and NSC-34 cells before and after SOD1^WT^ and SOD1^G93A^ incubation. The graphs show an increase of intracellular ROS in samples incubated with SOD^G93A^ in both cell lines compared to untreated cells; on the contrary, in the cells treated with SOD^wt^ a decrease in ROS due to the antioxidant activity of this enzyme. is observed (**Figures 5A,B**).

**FIGURE 6:**
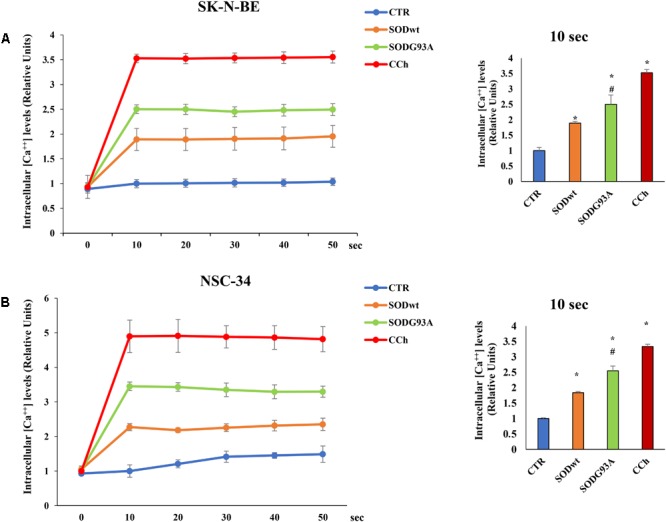
Effect of SOD1^wt^ and mutated SOD1^G93A^ on intracellular calcium levels in neuroblastoma SK-N-BE cells **(A)** and in NSC-34 cells **(B)**. The data are compared with control and with carbachol-induced intracellular calcium increase (positive control). The graphs on the left show the mean ± SEM values (*n* = 6) relative to control of the indicated time points. The histograms on the right show the values of 10 s point and the relative ANOVA analysis. ^∗^*p* < 0.001 vs. Ctr; ^#^*p* < 0.01 vs. SOD^wt^.

The effects of SOD1^wt^ and SOD1^G93A^ on intracellular calcium concentration in SK-N-BE and in NSC 34 cells, are shown in **Figures 6A,B**; as can be observed, the incubation with 400 ng/ml of SOD1^G93A^ significantly increases intracellular calcium concentration compared with wild type SOD1. The same trend was observed in NSC-34 cells incubated with the same amount of either SOD1^G93A^ or wild type SOD1; also, in this case, the calcium transients are noticeably increased by SOD1^G93A^ compared to SOD1^wt^.

**FIGURE 7:**
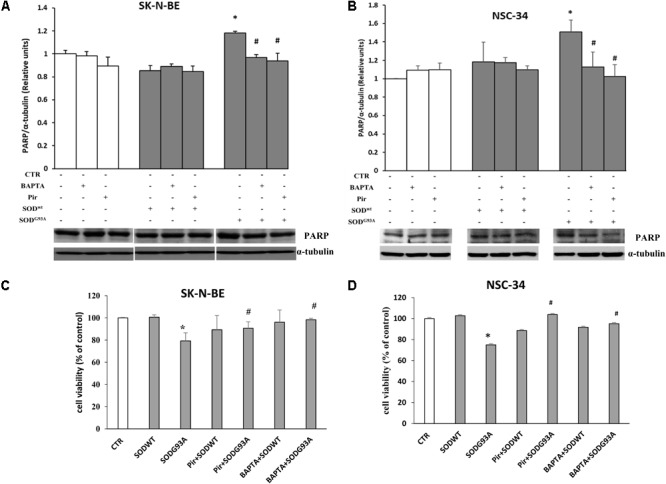
SK-N-BE and NSC-34 semiconfluent cells were preincubated with 15 μM of pirenzepine or with 10 μM of BAPTA-AM for 30 and 5 min respectively. Then, the cells were treated for 4 h with 400 ng/ ml of SOD1^wt^ or mutated SOD1^G93A^. Induction of apoptosis by SOD1^G93A^ on SK-N-BE **(A)** and NSC-34 **(B)** cells is showed by Western Blotting analysis of cleaved form of PARP-1 protein bands. Evaluation of cell viability by trypan blue assay in SK-N-BE and NSC-34 cells are showed in **(C,D)**, respectively; the data are reported as per cent variation compared to control. The data represent the means ± SEM relative to control obtained by densitometric analysis of cleaved PARP protein bands normalized to α-tubulin of three independent experiments. ^∗^*p* < 0.005 vs. Ctr; ^#^*p* < 0.005 vs. SOD^G93A^.

The results related to cytotoxicity and apoptosis, have been shown in **Figure 7**. The data shown in **(A,B)** demonstrate that in both cell lines incubated for 4 h with SOD1^G93A^, the cleaved form of PARP protein (89 KDa), an apoptosis marker, is statistically higher compared to control or SOD1^wt^ incubated cells. This effect is mediated by intracellular calcium level increase and by M1 muscarinic receptor since both the calcium chelator BAPTA and the specific M1 receptor inhibitor pirenzepine reverted the apoptotic effects of SOD1^G93A^. These data indicate that the overstimulation of M1 muscarinic receptor by the mutated SOD1 exerts, in addition to signaling function, a proapoptotic effect. These data are further confirmed by Trypan Blue assay experiments shown in **Figures 7C,D**.

## Discussion

We previously demonstrated that SOD1 interacts specifically with the cell surface of SK-N-BE by M1 muscarinic receptor, activating the cascade of reactions leading to the ERK1-2 and AKT phosphorylation and subsequent increase of intracellular calcium concentration.

It was also verified that M1 muscarinic receptor was widely expressed in different cell lines like SK-N-BE, MO3-13 and NSC-34

SOD1, as well as other proteins, are misfolded in familial and sporadic ALS, however it has not yet been established how this triggers the endoplasmic reticulum (ER) stress, associated with fragmentation of the Golgi apparatus and apoptosis. Roughly 20% of fALS is determined by SOD1 gene mutations (Neumann et al., 2006). It is also known that almost all SOD1 mutants maintain their enzymatic activity suggesting the existence of gain of toxic function rather than a loss of function (Strong et al., 2005; Dion et al., 2009).

(An unfolded protein response has been shown in spinal cord motor neurons of human patients with the sporadic form of ALS that is not restricted to SOD1 mutations (Atkin et al., 2008).

However, the relationship between the pathogenesis of familiar and sporadic forms of ALS has not yet been clarified, since in the latter, misfolded wild type-SOD1 protein activates the same neurotoxic pathway that is invoked by SOD1 mutants in familiar ALS (Bosco et al., 2010; Forsberg et al., 2010).

The mechanism by which mutated SOD1^G93A^ induces ER stress and Golgi apparatus fragmentation, and accumulation of misfolded or unfolded proteins at the endoplasmic reticulum lumen, has been extensively studied but is still unclear (Turner et al., 2005; Turner and Atkin, 2006; Nassif et al., 2010).

In this paper we showed that M1 muscarinic receptor was expressed in different cell lines like SK-N-BE, MO3-13 as well as in NSC-34.

Interestingly, we pointed out that, in both SK-N-BE and NSC cells, the mutated SOD1^G93A^ carried out a cytotoxic effect mediated by a more evident activation of ERK1-2 and AKT. These effects, involving M1 muscarinic receptor, are linked with a stronger increase of intracellular calcium levels compared to SOD1^wt^. In addition, the apoptotic role carried out by elevated intracellular calcium concentration induced by M1 receptor activation is confirmed by BAPTA–AM and pirenzepine experiments, respectively; in fact, in presence of calcium chelator and muscarinic M1 receptor antagonist, the SOD1^G93A^ mediated apoptotic effect, is reverted in both cellular lines. The more marked increase of intracellular calcium concentration, carried out by SOD1^G93A^, could represent a further inedited effect that leads to gain of function ascribed to this form of mutated SOD1. This last effect is also confirmed by cell viability experiments in which the incubation of NSC-34 and SK-N-BE cells with the SOD1^G93A^ determines apoptosis, evaluated by the cleaved form of PARP protein. Therefore, our results suggest that in some neurodegenerative diseases, like familiar amyotrophic lateral sclerosis (fALS), a gain of function due to mutated SOD1 could be ascribed to apoptosis mediated by raise of intracellular calcium concentration.

## Author Contributions

SD, PM, MS, and AS conceived and designed the experiments. RA, SD, LP, and AS performed the experiments. SD, PM, MS, and AS analyzed the data. AB, BD, MM, and LP contributed reagents, materials, and analysis tools. SD, PM, MS, and AS wrote the paper.

## Conflict of Interest Statement

The authors declare that the research was conducted in the absence of any commercial or financial relationships that could be construed as a potential conflict of interest.
